# Protective Effect of CXCR4 Antagonist DBPR807 against Ischemia-Reperfusion Injury in a Rat and Porcine Model of Myocardial Infarction: Potential Adjunctive Therapy for Percutaneous Coronary Intervention

**DOI:** 10.3390/ijms231911730

**Published:** 2022-10-03

**Authors:** Kai-Chia Yeh, Chia-Jui Lee, Jen-Shin Song, Chien-Huang Wu, Teng-Kuang Yeh, Szu-Huei Wu, Tsung-Chin Hsieh, Yen-Ting Chen, Huan-Yi Tseng, Chen-Lung Huang, Chiung-Tong Chen, Jiing-Jyh Jan, Ming-Chen Chou, Kak-Shan Shia, Kuang-Hsing Chiang

**Affiliations:** 1 Institute of Biotechnology and Pharmaceutical Research, National Health Research Institutes, Zhunan 35053, Taiwan; 2 Taipei Heart Institute, Taipei Medical University, Taipei 11031, Taiwan; 3 Department of Cardiology, Taipei Medical University Hospital, Taipei 11031, Taiwan; 4 Department of Internal Medicine, School of Medicine, College of Medicine, Taipei Medical University, Taipei 11031, Taiwan; 5 Graduate Institute of Biomedical Electronics and Bioinformatics, National Taiwan University, Taipei 106319, Taiwan

**Keywords:** CXCR4, ischemia, reperfusion, myocardial infarction, inflammation, endothelial progenitor cells, left ventricular ejection fraction, fibrosis, percutaneous coronary intervention

## Abstract

CXCR4 antagonists have been claimed to reduce mortality after myocardial infarction in myocardial infarction (MI) animals, presumably due to suppressing inflammatory responses caused by myocardial ischemia-reperfusion injury, thus, subsequently facilitating tissue repair and cardiac function recovery. This study aims to determine whether a newly designed CXCR4 antagonist DBPR807 could exert better vascular-protective effects than other clinical counterparts (e.g., AMD3100) to alleviate cardiac damage further exacerbated by reperfusion. Consequently, we find that instead of traditional continuous treatment or multiple-dose treatment at different intervals of time, a single-dose treatment of DBPR807 before reperfusion in MI animals could attenuate inflammation via protecting oxidative stress damage and preserve vascular/capillary density and integrity via mobilizing endothelial progenitor cells, leading to a desirable fibrosis reduction and recovery of cardiac function, as evaluated with the LVEF (left ventricular ejection fraction) in infarcted hearts in rats and mini-pigs, respectively. Thus, it is highly suggested that CXCR4 antagonists should be given at a single high dose prior to reperfusion to provide the maximal cardiac functional improvement. Based on its favorable efficacy and safety profiles indicated in tested animals, DBPR807 has a great potential to serve as an adjunctive medicine for percutaneous coronary intervention (PCI) therapies in acute MI patients.

## 1. Introduction

Cardiovascular disease (CVD) is the leading cause of death globally, with ischemic heart disease (IHD) accounting for 39–45% of these deaths [1]. Acute myocardial infarction (AMI) is the acute presentation of IHD and the main cause of death. In AMI, blood flow in the coronary arteries is interrupted, resulting in severe hypoxia of the myocardium that requires immediate and rapid treatment [2]. Reperfusion strategies are the current standard treatment for AMI. However, they may result in paradoxical cardiomyocyte dysfunction, known as ischemia-reperfusion injury (IRI), adding further damage to the myocardium [2,3]. Current reperfusion strategies mainly focus on vascular intervention procedures, such as percutaneous coronary intervention (PCI), to restore the blood flow timely, followed by pharmacological treatments using a thrombolytic, antiplatelet or anticoagulant agent(s) as an adjuvant medicine for arterial patency [4], but no effective therapeutic agents have been reported to protect vascular intervention from IRI [5]. Though underlying causes for reperfusion injury are complicated and unclear, several pathophysiologic events, including endothelial dysfunction, immune activation, inflammation, apoptosis and autophagy, free radical formation, etc., have been proposed for possible interpretations [2,6,7]. Among them, inflammation, mainly induced by neutrophil infiltration, is believed to be the key factor [8]. Additionally, the inflammatory response thus activated during myocardial ischemia can be exacerbated by further reperfusion injury during PCI. Chemokine–chemokine receptor networks, directing inflammatory cell movement to the site of tissue damage such as infarcted myocardium, have been well documented [9]. More specifically, C-X-C motif ligand 12 (CXCL12, also known as SDF-1α) expression increases in the infarct area after AMI or stroke, and then triggers the migration of CXCR4^+^ inflammatory cells [10,11,12] and CXCR4^+^ endothelial progenitor cells (EPCs) [13,14,15,16] to the lesioned sites. Notably, EPC recruitment may facilitate the post-infarct vascular repair in the area of ischemia [17,18,19,20,21,22]. Thus, regulating chemokine–chemokine receptor interactions along the CXCR4/CXCL12 axis, to reduce inflammation-induced tissue damage and enhance the recruitment of angiogenic cells (e.g., EPCs) to the infarcted region, may promote infarct healing. Indeed, several CXCR4 small-molecule antagonists, including AMD3100, TG-0054, POL6326 and POL5551, appear to meet these requirements and have been shown to enhance tissue repair and improve cardiac function after myocardial infarction as demonstrated in AMI animal models [10,11,23,24,25]. Herein, we wish to report that instead of continuous or multiple dosing of DBPR807, a single injection immediately prior to myocardial ischemia-reperfusion in AMI animal models (rats and mini-pigs) can reduce cardiac damage and promote functional recovery significantly, resulting in the desirable long-term prognosis. Results and discussion are presented as follows.

## 2. Results

### 2.1. Synthesis, Characterization and Biological Activity of DBPR807

Recently, studies have reported that DBPR807 (Appendix A) exhibited an excellent neuroprotective and anti-inflammatory effect against intracerebral hemorrhage (ICH) [11], and could potentiate kinase inhibitors (e.g., sorafenib) or checkpoint inhibitors (e.g., anti-PD-1) for hepatocellular carcinoma (HCC) treatment [26]. The synthetic procedure, structural characterization and preclinical profiles of DBPR807, including binding assay (IC_50_), chemotaxis functional assay (EC_50_), target specificity and patch-clamp assay, CYP450 inhibition, 67 off-target profiling, pharmacokinetic studies and non-GLP 14-day repeated-dose toxicology have been disclosed in above earlier works [11,26]. Inspired by previous reports on successfully applying CXCR4 antagonists, such as AMD3100 and TG-0054 [10,23,24,25], to AMI animal models, we speculated that DBPR807 should show better therapeutic efficacy in similar AMI settings in terms of its better pharmacological profiles.

### 2.2. DBPR807 Reduced Damage from H_2_O_2_-Induced Oxidative Stress In Vitro

**Figure 1:**
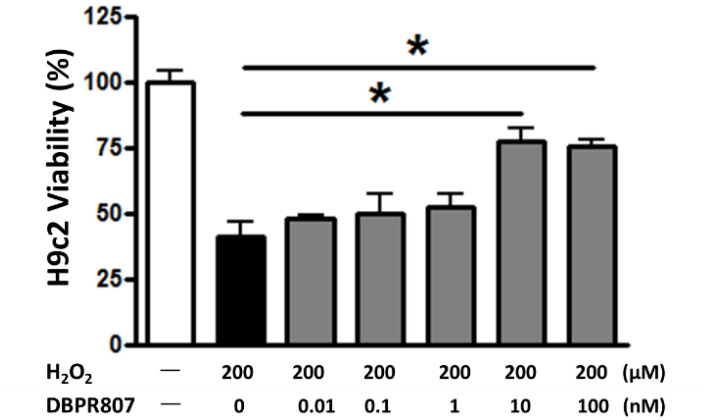
DBPR807 protected against H_2_O_2_-induced injury in H9c2 cells. H9c2 cells were pretreated by indicated concentrations of DBPR807, and then oxidative stress was induced with 200 μM H_2_O_2_. The data are presented as mean ± SEM. Statistical analysis was performed by *t*-test: * *p* < 0.05.

Previously, several studies have revealed that AMD3100 might attenuate the reactive oxygen species (ROS)-induced damage in vitro [27,28]. Similarly, our oxidative stress assay with DBPR807 (Figure 1) also showed that it could protect H9c2, the cardiomyocyte, against H_2_O_2_-induced injury at a low concentration (IC_50_ = 3.7 ± 0.6 nM) as effectively as the marketed CXCR4 antagonist AMD3100, implying that it may reduce cardiac damage, thus caused by ROS-induced inflammation.

### 2.3. DBPR807 Mobilized CXCR4-Expressing Stem/Progenitors Cells in Mice

**Figure 2:**
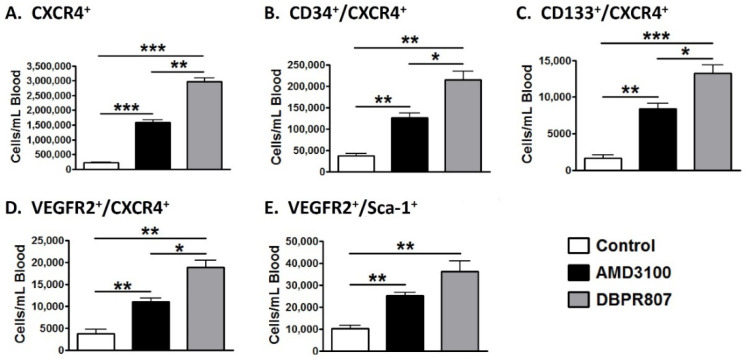
Various stem/progenitor cells were mobilized by DBPR807. Mice were subcutaneously injected with vehicle AMD3100 (6 mg/kg) or DBPR807 (20 mg/kg). At 2 h after dosing, the blood was harvested for analyzing the cell types of interest: (**A**) CXCR4^+^ cells, (**B**) CD34^+^/CXCR4^+^ cells, (**C**) CD133^+^/CXCR4^+^ cells, (**D**) VEGFR2^+^/CXCR4^+^ cells and (**E**) VEGFR2^+^/Sca-1^+^ cells. The data are presented as mean ± SEM (*n* = 3 per group). Statistical analysis was performed by *t*-test: * *p* < 0.05, ** *p* < 0.01 and *** *p* < 0.001.

Since DBPR807 is a typical CXCR4 antagonist, its stem cell-mobilizing ability is regularly tested to evaluate, particularly, the population of EPCs, which are considered to facilitate cardiovascular repair in infarcted heart [15,16]. DBPR807 was administered subcutaneously (SC) in 8- to 10-week-old C57BL/6 male mice. The CD34^+^/CXCR4^+^ (Figure 2B) and CD133^+^/CXCR4^+^ (Figure 2C), as well as VEGFR2^+^/CXCR4^+^ (Figure 2D) and VEGFR2^+^/Sca-1^+^ (Figure 2E) cell types, commonly recognized as biomarkers for EPCs, were analyzed through flow cytometry [29,30,31]. A maximal response dose (a plateau dose) of the positive control (AMD3100, 6 mg/kg) and DBPR807 (20 mg/kg) were given via injection SC, respectively. As a result, DBPR807 displayed more efficient capability than AMD3100 to move out various types of EPCs from the bone marrow to peripheral blood (Figure 2), supporting that it could be a potent stem cell mobilizer and qualifies for further efficacious testing. Nevertheless, we have to emphasize that the above EPC cell types of interest only account for ca. 9% of all CXCR4-expressing cells (Figure 2A); thus, whether other unidentified cell types could play a similar role as EPCs remains unclear and deserves to be further studied. The details of EPC counts in plots are provided in Appendix B.

### 2.4. DBPR807 Alleviated Cardiac Damage after IRI in Rats

**Figure 3:**
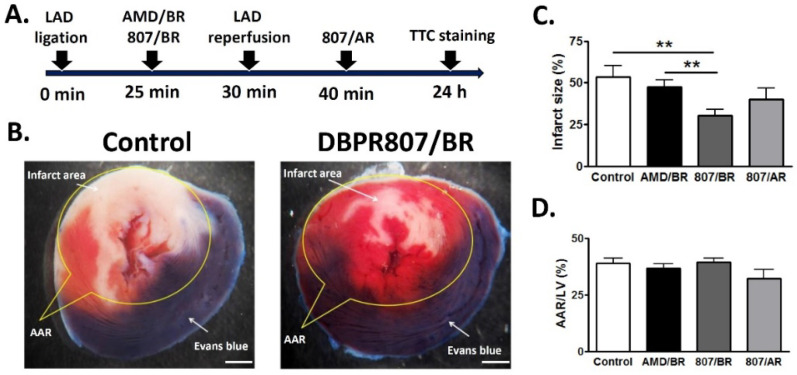
Infarct size assessment in rats. Rats were treated with vehicle AMD3100/BR (5 mg/kg, SC) or DBPR807/BR (5 mg/kg, SC) at 25 min after LAD artery ligation or DBPR807/AR (5 mg/kg, SC) at 40 min after LAD artery ligation (control, *n* = 10; AMD3100/BR or DBPR807/BR, *n* = 15; DBPR807/AR, *n* = 8). AAR and infarct size were evaluated 1 day after IR injury in heart via Evans blue and TTC-staining; AMD3100 (infarct area/AAR = 43%); DBPR807 (infarct area/AAR = 30%). (**A**) Treatment protocol. (**B**) Viable tissue is red, and the infarct area is white; scale bar = 1 mm. (**C**) Infarct size was determined by the infarct area/AAR ratio and quantified as a percentage. (**D**) AAR/LV area ratio was quantified as a percentage. The data are presented as mean ± SEM. Statistical analysis was performed by *t*-test: ** *p* < 0.01.

The experimental protocol (Figure 3A) depicted that the left anterior descending (LAD) artery of rats was transiently ligated for a 30-min ischemic period, and animals were administered subcutaneously with CXCR4 antagonists AMD3100 (5 mg/kg) or BBPR807 (5 mg/kg), respectively, at 5 min before reperfusion. Evans blue and TTC staining of cardiac tissue were performed 24 h following the surgically induced IR injury in the heart for analysis. Results demonstrated that rats treated with DBPR807 (5 mg/kg, *n* = 15) preserved far more viable tissue (red) than the control group (*n* = 10), as seen in Figure 3B. After quantitative analysis (Figure 3C), the ratio of infarct area to area at risk (AAR) for DBPR807 was significantly reduced to 30%, which was much lower than that of the control group (54%) and AMD3100 treatment group (43%), suggesting that DBPR807 produces a stronger protective effect against IRI than its marketed CXCR4 counterpart AMD3100 under the same setting.

**Figure 4:**
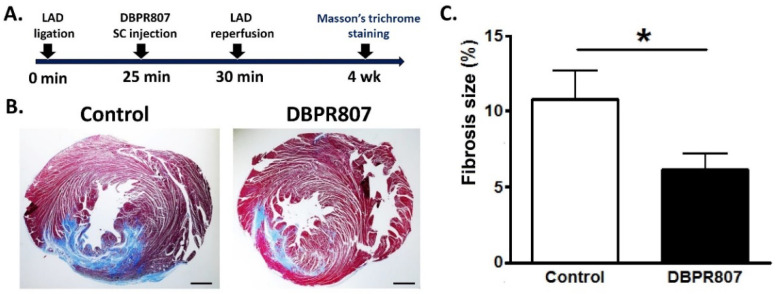
Fibrosis assessment in rats. Rats were treated with or without DBPR807 (5 mg/kg, SC) at 25 min after LAD artery ligation in heart (control, *n* = 15; DBPR807, *n* = 16). Fibrosis size was evaluated 4 weeks after IR injury via Masson’s trichrome staining. (**A**) Treatment protocol. (**B**) The fibrosis of tissue was stained in blue; scale bar = 1 mm. (**C**) The fibrosis size of the heart section from the rat was quantified as the ratio of fibrosis (blue) area to the left ventricle (LV) area. The data are presented as mean ± SEM. Statistical analysis was performed by *t*-test: * *p* < 0.05.

To our surprise, when DBPR807 was dosed twice instead, namely, before reperfusion (BR, 5 min) and after reperfusion (AR, 10 min), we found that the reduction in infarct size was as poor as that of a single-dose treatment after reperfusion (DBPR807/AR in Figure 3C), by which no statistically significant effect was observed compared to a single-dose treatment of AMD3100/BR. Moreover, when rats were sacrificed after 4 weeks following the similar treatment protocol (Figure 4A), DBPR807/BR could clearly reduce the fibrosis area (blue) as demonstrated with Masson’s trichrome stain of a heart section 4 weeks after IR injury (Figure 4B). Accordingly, quantitative analysis (Figure 4C) revealed that DBPR807/BR treatment could significantly shrink the fibrosis size to a smaller proportion (6% for the fibrosis area/left ventricle area, *p* < 0.05) in sharp contrast to that of the control group (11%), indicating that DBPR807 could reduce cardiac fibrosis and benefit the long-term prognosis. Notably, the survival rate of the control and DBPR807 groups were 76% and 84%, respectively, suggesting that there appeared to be a marginal improvement in the drug treatment group (*p* = 0.558).

### 2.5. DBPR807 Ameliorated Recovery of Cardiac Function after IRI in Mini-Pigs

**Figure 5:**
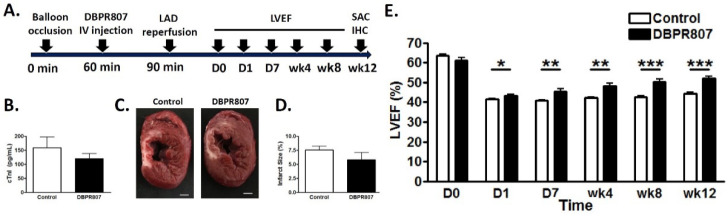
DBPR807 treatment recovers cardiac function after IR injury in the mini-pig myocardial infarction model. (**A**) Mini-pigs were treated with or without DBPR807 (3 mg/kg, IV) prior to balloon-induced IR injury (*n* = 10 per group) following the above experimental protocol. (**B**) Concentration of troponin I in blood after 24 h. (**C**) TTC staining after 12 weeks: viable tissue is red, and the infarct area is white; scale bar = 5 mm. (**D**) Infarct size was determined by the infarct area/LV area ratio and quantified as a percentage after 12 weeks. (**E**) Changes in LVEF (control vs. DBPR807) from D0 to 12 weeks were measured. The data are presented as mean ± SEM. Statistical analysis was performed by *t*-test: * *p* < 0.05, ** *p* < 0.01 and *** *p* < 0.001.

In addition to the above rat AMI model exerting a significant cardio-protective effect against IRI, a porcine AMI model, bearing a high similarity in coronary arterial structure and cardiac kinetics to human [32,33,34,35,36], was then established for further assessment of DBPR807. According to the experimental protocol shown in Figure 5A, a single dose of DBPR807 (3 mg/kg, IV) was injected 30 min before LAD artery reperfusion during 90-min balloon occlusion. As a result, troponin I was reduced by 25% in blood after 24 h (Figure 5B) and by 23% in infarct size after 3 months (Figure 5C,D). Though these data were not statistically significant, a trend of reducing cardiac damage was observed. More importantly, the left ventricular ejection fraction (LVEF), clinically representing the primary end point, was measured along the time points as scheduled in Figure 5A over 3 months. Initially, the LVEF of both control and DBPR807 treatment groups was worse than the baseline, wherein the LVEF dropped from 61.15 ± 2.69% and 63.53 ± 3.85% before surgery, respectively, to 39.98 ± 2.14% and 39.56 ± 3.58% after IRI. The observed LVEF (<40%) consistently, in both groups after myocardial infarction and reperfusion, reflected the similar extent of initial injury with manifestations combined for all forms of IRI, including myocardial stunning, reperfusion-induced arrhythmias, microvascular obstruction and myocardial reperfusion injury [5]. The interactions among these factors were dynamic and time-dependent, thus, it was extremely difficult to clarify the entire biochemical process. Serial changes in the LVEF for the DBPR807 treatment and control groups indicated that there was no noticeable difference in the LVEF between these two groups during 1-6 h after reperfusion. However, along an increase in the time axis, the LVEF of the DBPR807 treatment group was constantly improved and significantly higher than that of the control group as indicated below: 43.21 ± 1.93% vs. 41.38 ± 1.49% on day one (* *p* < 0.05), 45.54 ± 4.25% vs. 40.82 ± 1.65% on day seven (** *p* < 0.01); 48.12 ± 4.52% vs. 42.24 ± 1.62% on week four (** *p* < 0.01); 50.20 ± 4.67% vs. 42.46 ± 2.55% on week eight (*** *p* < 0.001); and 51.72 ± 4.14% vs. 44.23 ± 2.14% on week twelve (*** *p* < 0.001) as shown in Figure 5E. The corresponding echo data (M mode) are presented in Appendix C. Thus, the above findings strongly support that a single-dose treatment of DBPR807 at the onset of IRI may facilitate cardiac function recovery in a continuously increasing manner, and thereby benefit the long-term prognosis. A LVEF falling in between 50~70% was generally considered successful for cardiac function recovery. The survival rate of the control and DBPR807 groups were 53% and 59%, respectively, indicating that there appeared to be a marginal improvement in the drug treatment group (*p* = 0.709).

### 2.6. DBPR807 Preserved Density and Integrity of Capillary of Infarct Region after IRI in Mini-Pigs

**Figure 6:**
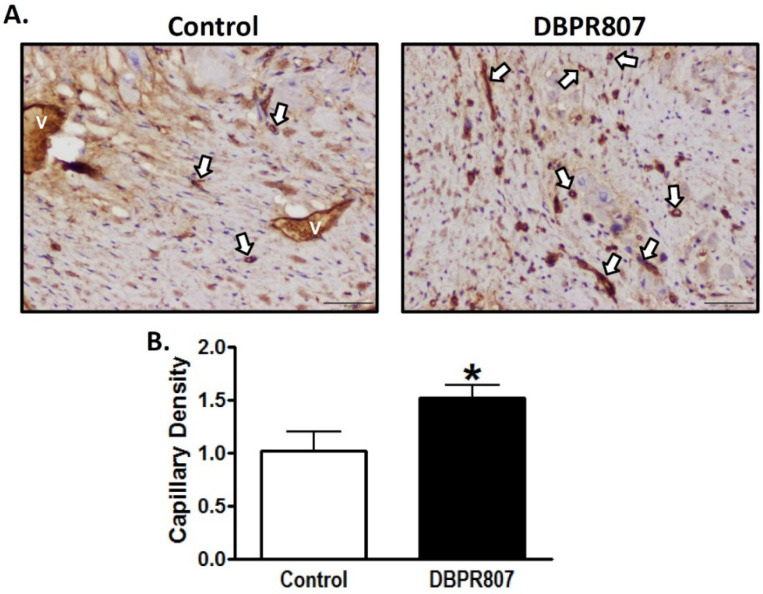
DBPR807 mitigated the reduction in capillary density after IR injury in the mini-pig model. Mini-pigs were treated with or without DBPR807 (IV, 3 mg/kg) after balloon-induced IR injury (*n* = 10 per group). (**A**) Isolectin B4 was stained 12 weeks after IR injury and capillaries are labeled in brown (white arrows); V represents vein; scale bar = 50 μm on 200× field. (**B**) The density of isolectin B4^+^ stain was quantified to determine capillary density. The data are presented as mean ± SEM. Statistical analysis was performed by *t*-test: * *p* < 0.05.

**Figure 7:**
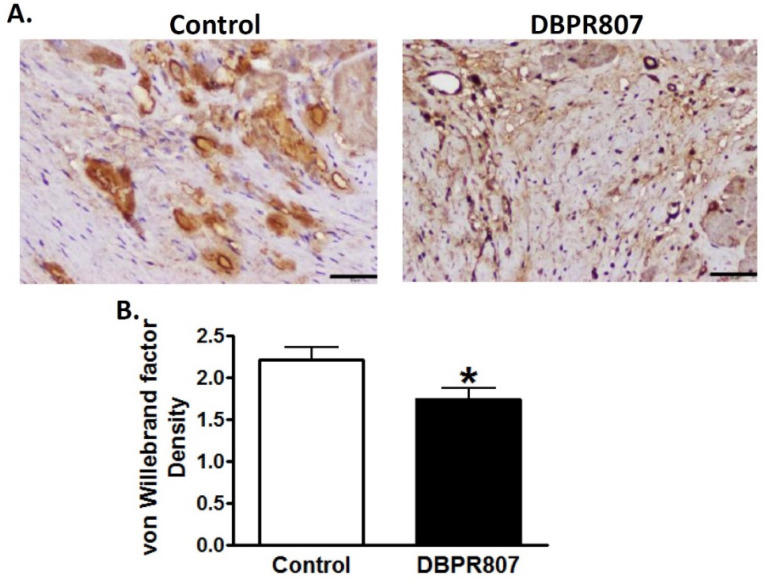
DBPR807 preserved integrity of capillaries after IR injury in the mini-pig model. Mini-pigs were treated with or without DBPR807 (3 mg/kg) after IR injury (*n* = 10 per group). (**A**) vWF was stained 12 weeks after IR injury; scale bar = 50 μm on 200× field. (**B**) The density of vWF was quantified and presented as mean ± SEM. Statistical analysis was performed by *t*-test: * *p* < 0.05.

To study the effect of DBPR807 to myocardial capillaries in the porcine IRI model, mini-pigs were sacrificed 12 weeks after transient induction of myocardial infarction followed by reperfusion (see experimental protocol in Figure 5A). Immunohistochemical staining with *Griffonia simplicifolia* isolectin B4 and von Willebrand factor (vWF) for infarct sections showed that DBPR807 treatment could significantly attenuate the decrease in capillary density (Figure 6, *p* < 0.05) and mitigate the leakage of capillaries (Figure 7, *p* < 0.05), indicating that DBPR807 may protect the complex capillary network and preserve the endothelium integrity. IRI affects more in endothelial cells than myocardial cells [37], IRI-related pathologies present tissue alterations in neovascularization, vascular integrity and capillary density via oxidative stress, inflammation, endothelial dysfunction and cell death [38]. Our data revealed that the early treatment of DBPR807 in IRI may induce mobilization of bone marrow-derived progenitor cells, thus, alleviating lesions caused by ischemia/reperfusion.

### 2.7. Pharmacokinetic Studies on DBPR807 following IV and Administration SC

Pharmacokinetic studies were investigated in mini-pigs following IV administration (Table 1), and in mice following injection SC, respectively. Results showed that both drug concentration (AUC = 37,845 ng/mL.h) and half-life (t_1/2_ = 4 h) in mini-pigs were much better than those in mice (AUC = 16,499 ng/mL.h; t_1/2_ = 1 h), implying that clinically, DBPR807 might have desirable blood exposure and promising therapeutic effects because porcine physiological conditions are more close to humans.

## 3. Discussion

AMI is one of the leading causes of death in the modern society, and PCI is considered as an effective standard medical procedure to rescue patients with AMI. However, reperfusion of the occluded arteries during PCI may further exacerbate the ischemic myocardium. As such, how to prevent IRI thus caused could be the most challenging issue to be addressed. This study aimed to report that DBPR807 may serve as a potential adjunctive therapeutic agent when PCI is performed. EPC recruitment, considered/hypothesized to facilitate tissue repair and healing, has been well documented [13,14,15,16,17,18,19,20,21,22,23]. Based on this argument, analyzing the stem cell mobilization profiling has been typically used as a tool to screen any potential CXCR4 antagonists for further efficacious studies in different disease animal models [39,40,41]. Accordingly, DBPR807 exhibiting good ability to mobilize EPCs with biomarkers, such as CD34^+^/CXCR4^+^ and CD133^+^/CXCR4^+^ (Figure 2), was then selected to test its IRI-protective effects in AMI animals. As a screening tool, using healthy mice rather than diseased ones to identify potential compounds for further studies is quite common in that research time and can could be greatly cut down. In addition, since EPCs are originated from bone marrow rather than the heart, it is reasonable to propose that EPC-mobilizing ability should be highly similar in both healthy and IRI/AMI animals. Furthermore, whether EPC recruitment is responsible for reducing tissue damage remains a hypothesis. Indeed, EPC counts only account for ~9% of all BM-derived stem cells mobilized by DBPR807 (Figure 2), implying that other unidentified CXCR4^+^ cells, such as MSCs [24], might also contribute to IRI-protective effects. Our studies on DBPR807 mainly revealed that no significant efficacy was observed in AMI rats as the repetitive-dose treatment, following administration SC before and after reperfusion, was adopted; however, when it was treated at a single high dose before reperfusion, a significant effect could be produced. These results imply that mechanistically, DBPR807/BR might play a unique dual-function role, not only for suppressing ROS-induced inflammation (Figure 1) in the lesioned site caused by reperfusion timely, but also for blocking the CXCR4/CXCL12 axis transiently to recruit EPCs from bone marrow (Figure 2) for subsequent repair of the ischemic myocardium. We strongly suggest that suppressing reperfusion-induced inflammation efficiently at a very early stage should be crucial for the significant reduction in infarct and fibrosis size observed in rats (Figure 3 and Figure 4), as well as the recovery of cardiac function (Figure 5) in mini-pigs. Above inference might also shed light on why CXCR4 antagonists should be treated at a high single dose before reperfusion rather than using multiple or continuous doses after reperfusion, because the latter approaches might impair/block the EPC recruitment to the lesioned sites.

Interestingly, during the preparation of the manuscript, there was a report by Zhang, et al. [42], stating that when a vascular leakage blocker, known as CU06-1004, was delivered before reperfusion in a single high dose in rats, it could significantly protect vascular integrity and improve cardiac function by suppressing reperfusion-induced inflammation. Moreover, no in vivo efficacy was observed when the repetitive low-dose treatment was adopted for CU06-1004. Similarly, we found that DBPR807 treated at a single high dose in the porcine AMI model before reperfusion could preserve vascular/capillary density (Figure 6) and integrity (Figure 7) of infarcted regions remarkably, leading to a significant improvement in cardiac function recovery (Figure 5). Despite above promising findings however, the exact molecular target or biomarker responsible for IRI remains to be determined and becomes an important part of our future work.

In conclusion, the favorable efficacy and safety profiles of DBPR807 may make it become a valuable add-on to the PCI therapy in AMI patients. Instead of traditional continuous or multiple-dose treatment at different intervals of time, a single-dose treatment of CXCR4 antagonists at an appropriate time point before reperfusion is suggested to produce a maximal improvement in the cardiac function recovery in MI therapies (e.g., PCI).

## 4. Materials and Methods

### 4.1. Animals and Material

Male C57BL/6 mice and SD rats were purchased from the National Laboratory Animals Center (Taipei, Taiwan, R.O.C.). The animals received human care in compliance with the “Guide for the Care and Use of Laboratory Animals” published by the National Academy of Sciences, and the experimental procedures and protocols were approved by the Institutional Animal Care and Use Committee (IACUC) (IACUC approval No: 105094) of National Health Research Institutes (NHRI) (Miaoli, Taiwan). Lee-Sung mini-pigs were obtained from the Agricultural Technology Research Institute (ATRI) (Miaoli, Taiwan) and the experimental processes of mini-pig were approved by the IACUC of ATRI (IACUC approval No: 108062C3). The (3-{4-[2-({4-[3-(3-Cyclohexylamino-propylamino)-propyl]-furan-2-ylmethyl}-amino)-6-methyl-pyrimidin-4-ylamino]-piperidin-1-yl}-3-oxo-propylamino)-acetic acid hydrochloride salt (DBPR807) was synthesized according to Song et al. 2021 [26].

### 4.2. Cell Culture

The rat H9c2 cells, obtained from the ATCC, were maintained in Dulbecco’s modified Eagle’s medium (DMEM) (Gibco, New York, NY, USA) at 37 °C in an incubator (Thermo Fisher Scientific, New York, NY, USA) with an atmosphere of 5% CO_2_. The culture media were supplemented with 10% heat-inactivated fetal bovine serum (FBS) and 1% penicillin and streptomycin (HyClone, Logan, UT, USA).

### 4.3. H_2_O_2_-Induced Damage of Cardiac Myoblast Cells

H9c2 cells, derived from rat cardiac myoblasts, were seeded in a 96-well plate at a density of 2 × 10^4^ cells/well. After 24 h, cells were pretreated with indicated concentrations of DBPR807 for 1 h, then cell death was induced by treatment with 200 μM H_2_O_2_. After additional 24 h, 10 μL PrestoBlue cell viability reagent (Invitrogen, Waltham, MA, USA) was added to each well. The plates were incubated at 37 °C in 5% CO2/95% humidified air for 1.5 h and then fluorescence at excitation:560/emission:590 was read to measure cytotoxicity with VICTOR2 (PerkinElmer, Waltham, MA, USA).

### 4.4. Rat Model of Reperfused AMI

The male Sprague Dawley rat (400–500 g) was weighed and then anesthetized with isoflurane (3%). An endotracheal tube was inserted into the trachea with assistance using an otoscope and coronary guidewire. Endotracheal intubation was then performed to create the mechanical ventilation by connecting the endotracheal tube to a Kent scientific ventilator. After intubation was completed, the fur around neck and chest areas was shaved using an electric shaver. Then, the surgery area was scrubbed and disinfected with 10% povidone iodine solution, followed by wiping the area with 70% alcohol. The chest of the anesthetized animal was opened at the left fourth intercostal space and then a chest retractor was positioned within the intercostal space to expose the left ventricle of the rat heart. The pericardium was then opened, and the position of the left anterior descending (LAD) artery was identified. The LAD artery was transiently ligated using a 6-0 nylon suture tied up on a PE ring for a 30-min ischemic period. To allow cardiac reperfusion, a surgical blade was used to cut the knot in the ligature and the chest retractor was removed. At 5 min before or 10 min after reperfusion, the animal was administered subcutaneously with saline or CXCR4 antagonists (5 mg/kg AMD3100 or DBPR807). The sternal incision and skin wound was closed with a 6-0 nylon suture with an interrupted suture pattern. Excess air in the pleural cavity was drawn out by using a scalp vein tube to prevent the pneumothorax. After the surgery was completed, the isoflurane supply was terminated, and the ventilator air supply was kept for at least 10 min. Once the animal’s spontaneous breath was restored, the endotracheal tube was gently removed, and 0.1 mg/kg of buprenorphine was administered immediately after surgery. Then, the animal was placed in an animal cage and allowed to recover from anesthesia.

### 4.5. Histological Evaluation of the Infarct Size by TTC Staining in Rats

Following completion of the 24-h reperfusion period, the rat was anesthetized again with 3% isoflurane and the LAD artery was ligated once again. An amount of 2 mL of 5% Evans blue was injected into the tail vein and allowed to perfuse for 2 min to define the ischemic region. The rat heart was then rapidly excised and washed with saline. The heart was frozen at −80 °C and cut into 2 mm thick sections. Slices were then incubated in 1% 2,3,5-triphenyltetrazolium chloride (TTC) solution for 10 min at 37 °C and fixed in 10% formalin for 5 min to determine the infarct necrotic area. Images of each slice were captured by a digital camera and the infarct size was measured using Image Pro Plus software. Infarct size was determined by (sum of infarct necrotic areas (white region) from all sections)/(sum of ischemic areas (white and red regions) from all sections) × 100. The ischemic area was defined as the myocardial AAR.

### 4.6. Histological Evaluation of the Fibrosis Size by Masson’s Trichrome Staining in Rats

One month after the cardiac ischemia-reperfusion surgery, the rat was anesthetized again with 3% isoflurane. The rat heart was then rapidly excised and washed with saline. The heart was frozen at −80 °C and cut in a semi-frozen state into 2 mm thick sections. Slices of rat heart of 2 mm thickness were collected and fixed with 10% buffered neutral formalin solution. Specimens were embedded in paraffin and sectioned at a thickness of 5 μm via a microtome. Each specimen was stained with Masson’s trichrome stain for determination of the fibrosis area. Images of each slice were captured by a digital camera and the fibrosis area was measured using Image Pro Plus software. Fibrosis size was determined by (sum of fibrosis areas (blue region) from all sections)/(left ventricle from all sections) × 100.

### 4.7. Mini-Pig Model of Reperfused AMI

The Lee-Sung mini-pig (25–32 kg) was weighed and anesthetized by intramuscular injection of a mixture of Zoletil 50 (6 mg/kg) and Rompun (2.2 mg/kg) prior to surgery. The animal was placed on the operating table in a supine position and the trachea was intubated using a laryngoscope and a tracheal catheter, which was fixed to the palate with a permeable adhesive tape. Then, the animal was endotracheal intubated and connected to a veterinary ventilator (CMS, mini Vent 3) for controlled ventilation in the VCV mode (volume control monitoring). Anesthesia was maintained over the whole surgical procedure with 2% isoflurane. Venous access was obtained by cannulating the ear vein with a 24 G IV cannula. An amount of 25 mg/kg of Cefazolin was given to prevent infections. The surgical areas were disinfected with iodine 2% and sterile surgical drapes were used to cover the nonsterile parts of the pig. Lidocaine (3 mg/kg) was injected subcutaneously for local anesthesia in the surgical area. A medial incision was performed in the neck. The linea alba was passed to minimize muscle damage and the carotid artery and internal jugular vein next to the trachea were bluntly approached. Arterial access was achieved by cannulating the internal carotid artery with a 5.5F sheath using the Seldinger technique. The sheath was fixed to the artery, making sure the artery was not fully occluded by the suture. An amount of 200 IU/kg heparin was administered immediately after inserting the sheaths to inhibit thrombus formation. A second dose of 200 IU/kg heparin was administered to the pig at the 30th min of the procedure to maintain cardiovascular heparinization. A 5.5F guiding catheter was inserted via the previously placed sheath into the left carotid artery. It was advanced to the left coronary artery to reach the left anterior descending (LAD) coronary artery under X-ray guidance. Contrast medium was injected using a 5 mL syringe via the guiding catheter to perform a baseline coronary angiography. Fluoroscopic guidance was used to advance the PCI catheter until it reached approximately the middle of the LAD artery and then the pressure in the inflation device was increased to 2 atm to inflate the balloon of the PCI catheter and induce myocardial ischemia for 90 min. An amount of 3 mg/kg DBPR807 or control (saline) was injected intravenously after 60 min of induced myocardial infarction. After 90 min of induced myocardial infarction, the PTCA balloon was slowly deflated, and post-infarction echocardiography was performed to record LVEF changes. The PTCA balloon and guide catheter were removed. The introducer(s) was removed and the artery was ligated. The surgical incision site(s) was closed in layers. During recovery from anesthesia, floor mats and warming lamps were given to avoid loss of temperature. Oral pain relief tablet Carprofen (2–4 mg/kg) was given for 3 consecutive days for pain relief.

### 4.8. Determination of Blood Cardiac Troponin I in Mini-Pigs

At 24 h after reperfusion, the serums were centrifuged at 1000 g at 4 °C for 10 min. The serums were analyzed to determine the cardiac troponin I level using an ELISA kit (ACE Biolabs, Taoyuan, Taiwan, ROC).

### 4.9. Histological Evaluation of the Infarct Size by TTC Staining in Mini-Pigs

At the end of the 12-week test, the pig was euthanized after anesthesia, and the hearts were taken out for TTC staining and recording of myocardial necrosis areas. Images of each section (10–15 mm thick) were captured by a digital camera and the infarct size was measured using Image J software. Infarct size was determined by (sum of infarct necrotic areas (white region) from all sections)/(sum of LV regions from all sections) × 100.

### 4.10. Determination of the LVEF in Mini-Pigs

The left ventricular ejection fraction (LVEF) was measured using the ACUSON Antares ultrasound system (Siemens, San Jose, CA, USA). The measurements were performed at defined time points (basal (before surgery), 1 h, 3 h, 6 h, 24 h, 1 week, 2 weeks, 4 weeks, 8 weeks and 12 weeks after reperfusion).

### 4.11. Histological Evaluation of the Capillary Density and Integrity in Mini-Pigs

Specimens were fixed in 10% formalin (*v*/*v*) for at least 24 h, then embedded in paraffin and sectioned at a thickness of 5 μm via a microtome. The sections were stained with anti-von Willebrand factor (ab6994 at 1/3200, Abcam, Waltham, MA, USA) or biotinylated isolectin B4 (B-1205 at 10 μg/mL, Vector lab, Newark, CA, USA) and observed with an inverted microscope (IX83, Olympus, Japan). The grading was as follows: 0: no positive; 1: clear decrease with some positivity remaining; 2: minimal decrease in staining; 3: no loss of staining.

### 4.12. Pharmacokinetic and Toxicological Studies

Male C57BL/6 mice, each weighing 23.4–25.4 g, were quarantined for 1 week before drug treatment. DBPR807 dissolved in 100% saline was individually given to mice (*n* = 3) subcutaneously (6 mg/kg; nonfasted mice). Blood samples were collected via cardiac puncture at defined time points (0 (immediately before dosing), 0.03, 0.08, 0.25, 0.5, 1, 2 and 4 h after dosing) and then stored at −80 °C. The volume of the dosing solution given was 100 μL for each mouse. The plasma samples were analyzed with liquid chromatography–tandem mass spectrometry (LC–MS/MS), and the data were calculated by a standard noncompartmental method using the Kinetica software program (InnaPhase).

### 4.13. Flow Cytometry Analysis

C57BL/6 male mice were treated with potential CXCR4 antagonist individually by subcutaneous injection, and then blood samples containing mobilized stem/progenitor cells were collected 2 h later. After being labeled with specific antibodies (purchased from eBioscience, San Diego, CA, USA), including FITC-conjugated anti-CD34 (clone RAM34), PE-conjugated anti-CD133 (clone 13A4), PE-conjugated anti-VEGFR2 (clone Avsa12a1), APC-conjugated anti-Sca-1 (clone D7) and APC-conjugated anti-CXCR4 (clone 2B11), cells were washed, characterized and quantified using a flow cytometer (Guava Technologies, Hayward, CA, USA). Each data point included at least 60,000 events for measurement of various cell types.

### 4.14. Statistics

All statistical analyzes were performed with Graph Pad Prism software. According to the data distribution, the *t*-test was used. *p*-values smaller than 0.05 were considered to indicate statistical significance for all tests.

## 5. Patents

The following global patents have been granted for DBPR807: TWI664174; US10882854; JP6982716; AU2018208366; CA3047164; NZ754272; RU2756055C2; KR102335082; IN379503.

## Figures and Tables

**Table 1:** Pharmacokinetics of DBPR807 in mice and mini-pigs.

DBPR807	T1/2 (h)	AUC (0-Inf) (ng/mL × h)
6 mg/kg, SC, Mice ^a^	1.0	16,499 ± 878
3 mg/kg, IV, Mini-Pig ^b^	4.0	37,845 ± 3745

^a^ Values are presented as mean ± SD (*n* = 3) following injection SC in C57BL/6 mice, ^b^ Values indicate mean ± SD (*n* = 3) following IV in Lee-Sung mini-pigs.

**Figure A1:**
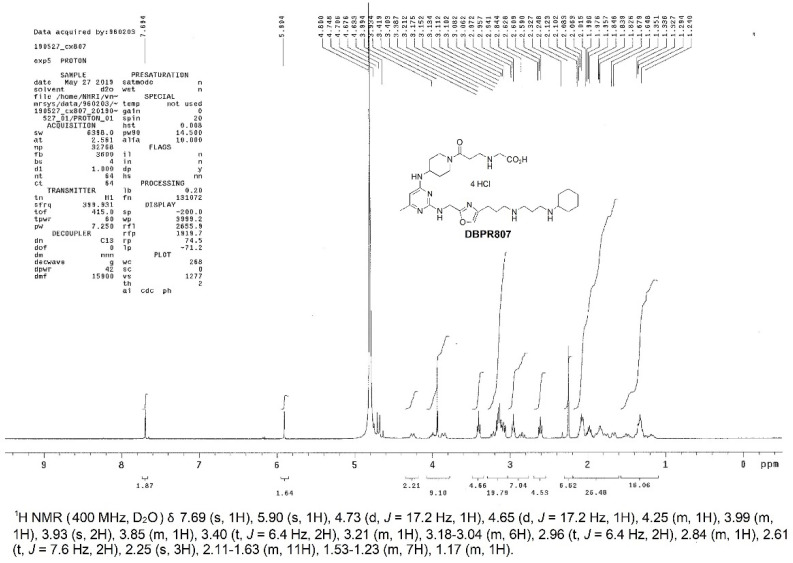
Structural characterization of DBPR807 with ^1^H NMR spectral analysis.

**Figure A2:**
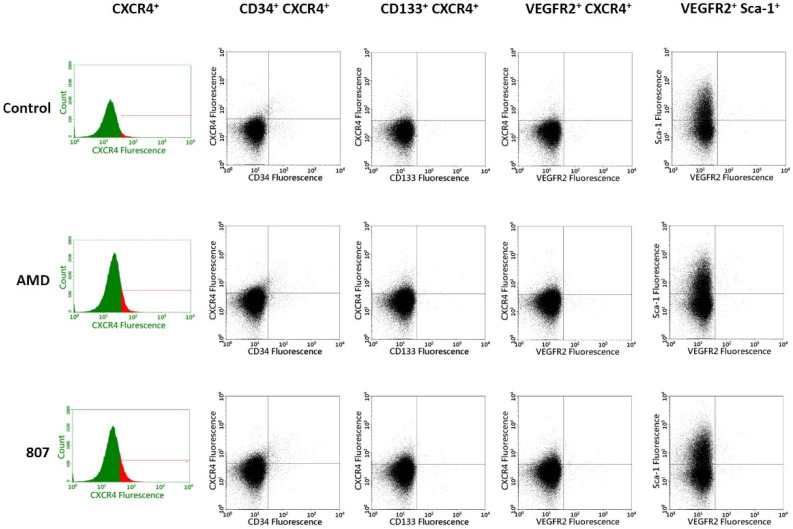
Gating strategies and FACS plots of AMD3100 and DBPR807.

**Figure A3:**
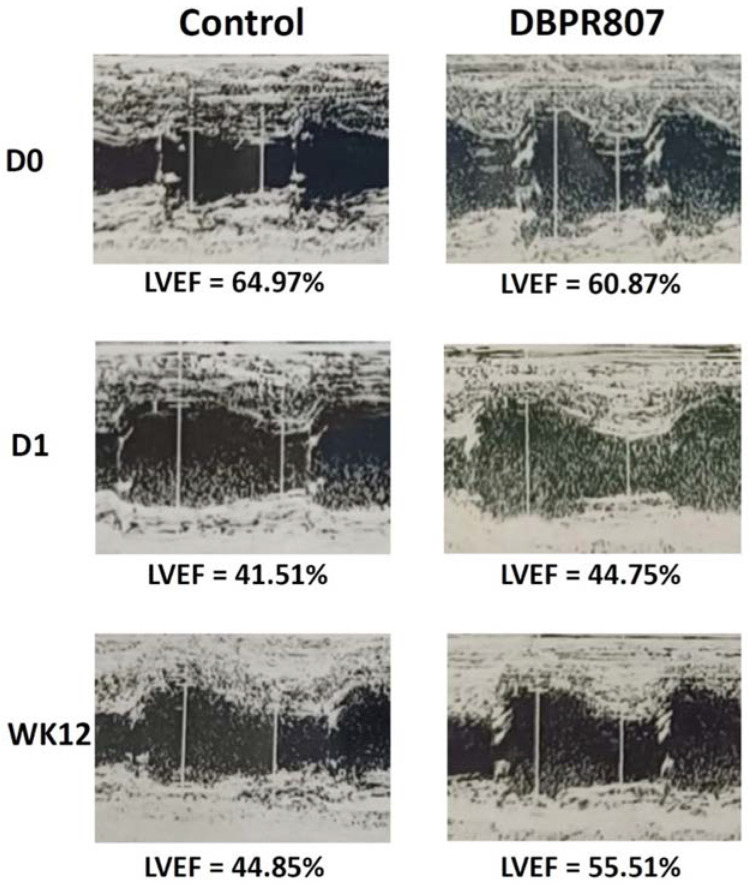
Effects of DBPR807 on the LVEF measured with echo in mini-pigs.
